# Microencapsulation of *Laurus nobilis* L. Leaf Extract in Alginate-Based System via Electrostatic Extrusion

**DOI:** 10.3390/foods12173242

**Published:** 2023-08-28

**Authors:** Erika Dobroslavić, Zoran Zorić, Verica Dragović-Uzelac, Ivona Elez Garofulić

**Affiliations:** 1 Department of Food Engineering, Faculty of Food Technology and Biotechnology, University of Zagreb, Pierottijeva 6, 10 000 Zagreb, Croatia; edobroslavic@pbf.hr (E.D.); vdragov@pbf.hr (V.D.-U.); 2 Centre for Food Technology and Biotechnology, Faculty of Food Technology and Biotechnology, University of Zagreb, Petra Kasandrića 3, 23 000 Zadar, Croatia; zzoric@pbf.hr

**Keywords:** laurel, polyphenols, stability, antioxidant activity, bioavailability, release kinetics

## Abstract

Bay leaves (*L. nobilis* L.) are a rich source of polyphenols that hold great potential for application in functional food products in which where the main challenges are the polyphenols’ low stability and bioaccessibility, which can be overcome through different microencapsulation techniques, such as electrostatic extrusion, which hasn’t been applied for the encapsulation of bay leaf polyphenols (BLP) to date. Therefore, the main goal of this research was to evaluate the potential of this technique through monitoring the polyphenolic content, antioxidant activity, release kinetics, and bioaccessibility of the encapsulated BLP. The results showed that electrostatic extrusion was suitable for the encapsulation of BLP, where 1% alginate and 1.5% CaCl_2_ with 0.5% chitosan resulted in the highest encapsulation efficiency (92.76%) and antioxidant activity in vitro. The use of 1.5% or 2% alginate with 5% CaCl_2_ + 0.5% chitosan showed the most controlled release of polyphenols, while encapsulation generally increased the bioaccessibility of BLP. The results showed that electrostatic extrusion can be considered an efficient technique for the microencapsulation of BLP.

## 1. Introduction

Bay leaf (*L. nobilis* L.) is a shrub widely distributed in the Mediterranean area, where its leaves have been traditionally used in cuisine and folk medicine for treating various gastrointestinal and respiratory health problems due to the beneficial effects of the bioactive molecules present in this plant part. Polyphenols, including flavonoids (mainly quercetin and kaempferol glycosides), phenolic acids, and proanthocyanidins, are largely responsible for these beneficial effects due to their antioxidative, antimicrobial, cardioprotective, neuroprotective, antiproliferative, and anti-inflammatory properties [1]. The main obstacle in an efficient utilization of these valuable properties is the tendency of polyphenols to degrade during storage under different temperatures, humidity, light, and pH [2], as well as their low bioavailability (degree of absorption in the gastrointestinal tract) related to their low bioaccessibility (release from food matrix), instability during digestion in the gastrointestinal tract, and difficult cell membrane diffusion [3]. Microencapsulation via different techniques has emerged as a concept which can be applied in order to overcome the mentioned shortcomings and allow the application of polyphenols in different functional products. To the best of the authors’ knowledge, encapsulation of BLP was investigated only in two studies [4,5], where it was suggested that BLP can be effectively encapsulated by spray-drying and nano-liposome encapsulation. Further research on these and other encapsulation techniques focusing on the optimization of their parameters with the goal of achieving maximum yields, stability, and bioavailability is a key step in the development of new or enhanced functional products utilizing the maximum potential that BLP holds. Electrostatic extrusion is a microencapsulation technique based on passing a biopolymer (most often sodium alginate) through a nozzle into a cross-linking (gelling) solution with the use of an electric field, resulting in uniform beads [6]. This technique is suitable for the microencapsulation of both hydrophilic and lipophilic compounds and has been applied for the encapsulation of polyphenols from different herbs [7,8]. It therefore holds the potential to be an efficient tool for preserving the quality and enhancing the bioavailability of BLP. Alginate is a water-soluble linear anionic marine polysaccharide composed of β-D-mannuronate and α-L-guluronate residues linked by 1–4 glycosidic bonds which forms gel in the presence of polyvalent ions, among which Ca^2+^ is the most suitable since it results in non-toxic and biocompatible complexes through a relatively cheap and simple process [6]. The encapsulation efficiency (EE) and preservation of biological activity of polyphenols encapsulated in a calcium–alginate complex may be enhanced by adding other biopolymers, such as chitosan, which is a non-toxic and biocompatible cationic polysaccharide built by N-acetyl-D-glucose-amine and D-glucosamine residues linked by 1–4 glycosidic bonds with the ability to form stable complexes with other anionic crosslinking agents [6]. Moreover, the addition of chitosan may result in a more controlled release of polyphenols [9] from the polymeric complex as well as increase their bioavailability [10]. Research on bioavailability for use in functional foods and dietary supplements is highly relevant since the concentration of polyphenols is not necessarily proportional to bioavailability [11]. However, it is often challenging due to the complexity of human physiology and ethical issues. Therefore, in vitro bioaccessibility assessment methods are often applied since they are relatively fast, simple, inexpensive, repeatable, and representative of data from in vivo studies, thus allowing a more efficient product formulation [12]. 

Since electrostatic extrusion has not been applied for the microencapsulation of BLP to date, the objective of this study was to evaluate the potential of this technique by varying the encapsulation parameters (percentage of sodium alginate, content of CaCl_2_, and presence of chitosan in the cross-linking (gelling) solutions) and monitoring the total and individual polyphenolic contents, antioxidant activity, release kinetics, and bioaccessibilities of selected polyphenols in the obtained beads. The results of this work will widen the scarce knowledge on the microencapsulation of BLP, making a step toward industrial utilization of this valuable plant material. In addition, insight into the influence of different electrostatic extrusion parameters on individual polyphenolic compounds will be provided which will offer valuable information for application on polyphenolic extracts from other plant materials as well.

## 2. Materials and Methods

### 2.1. Chemicals and Reagents

Milli-Q system (Millipore, Bedford, MA, USA) was used for distilled water purification. Folin–Ciocalteu reagent, sodium carbonate, sodium bicarbonate, calcium chloride, sodium acetate, formic acid (98–100%), and FeCl_3_ × 6H_2_O were procured from Kemika d.o.o. (Zagreb, Croatia). Methanol (99.8%), ethanol (96%), and anhydrous sodium citrate were purchased from from Lach-ner d.o.o. (Neratovice, Czech Republic). HPLC grade acetonitrile was procured from J.T. Baker Chemicals (Deventer, the Netherlands). Acros Organics B.V.B.A. (Thermo Fisher Scientific, Geel, Belgium) supplied TPTZ and Trolox. Glacial acetic acid, hydrochloric acid (37%), DMSO, DPPH, low viscosity sodium alginate, chitosan from shrimp shells (≥75% deacetylated), pepsin (≥500 U/mg, from porcine gastric mucosa), bile salts, and pancreatin (4 × USP, from porcine pancreas) were procured from Sigma-Aldrich Chemie GmbH (Steinheim, Germany). Sigma-Aldrich Corporation (St. Louis, MO, USA) provided myricetin, quercetin-3-glucoside, gallic, caffeic, ferulic, syringic, protocatechuic, rosmarinic, chlorogenic, and *p*-coumaric acid authentic standards. Extrasynthese (Genay, France) provided authentic standards of rutin, epicatechin gallate, catechin, epigallocatechin gallate, luteolin, apigenin, procyanidin B2, and kaempferol-3-glucoside. The stock solutions of standards were prepared in methanol (ethanol–0.5% *v*/*v* DMSO for apigenin) and diluted to five concentrations of working standard solutions.

### 2.2. Plant Material

A dry commercial sample of bay leaves collected in November 2021 in Lovran, Croatia was procured from Šafram d.o.o. (Zagreb, Croatia) and stored in a paper box at room temperature. The leaves were ground into coarse powder prior to the extraction using an electric grinder (OmniBlend V Blender 1200 W, VerVita, Croatia). Drying to constant mass at 105 °C [13] was used to determine leaf dry matter (>95%).

### 2.3. Extract Preparation

The extraction was performed on YC-010 5 L multi-functional extracting tank (Pilotech, Shanghai, China). Briefly, the ground bay leaf sample was mixed with distilled water in the extracting tank at a sample:solvent ratio of 1:7.5 and subjected to extraction for 10 min at the pressure of 0.07 MPa and temperature of 70 °C. The extract was collected and filtered through Whatman No. 40 filter paper (Whatman International Ltd., Kent, UK) into 1 L volumetric flask, made up to volume with distilled water, and transferred to a glass bottle which was stored in the refrigerator at 4 °C. The extract’s dry matter (2.13%) was analyzed by drying to constant mass at 105 °C [13].

### 2.4. Total Phenolic Content (TPC)

The total phenolic content (TPC) of the bay leaf extract was determined according to a previously described spectrophotometric method [14]. Briefly, 100 µL of extract (solvent for blank), 200 µL of Folin–Ciocalteu reagent, 2 mL of distilled water, and 1 mL of Na_2_CO_3_ were mixed in a reaction tube and incubated at 50 °C for 25 min, and the absorbance was read at 765 nm on a VWR UV-1600PC Spectrophotometer (VWR, Wayne, PA, USA) with gallic acid as standard. All samples were analyzed in duplicate and the TPC was expressed as mean value of gallic acid equivalents (GAE) in mg per g of leaf (bead) ± standard deviation.

### 2.5. Electrostatic Extrusion

For the electrostatic extrusion, alginate solutions (1.0, 1.5 and 2% *w*/*v*) were prepared by dissolving the adequate mass of low viscosity alginate in 100 mL of the bay leaf water extract (distilled water for the blank solutions) and stirring overnight at room temperature. Six different gelling solutions were prepared. The 1.5, 3, and 5% *w*/*v* calcium chloride solutions (pH 7) were produced by dissolving the adequate mass of calcium chloride in 1000 mL of distilled water. The calcium chloride solutions containing 0.5% *w*/*v* chitosan (pH 4) were prepared as previously described [15], with modifications. Briefly, an appropriate amount of chitosan was dissolved in 1% *v*/*v* acetic acid. Afterwards, adequate amounts of calcium chloride (1.5, 3 and 5% *w*/*v*) were added, and the solutions were made up to volume with 1% *v*/*v* acetic acid in a 1000 mL volumetric flask. The encapsulation was performed on Büchi Encapsulator B-390 (Büchi, Switzerland) with a 1 mm nozzle at following fixed parameters: pressure 0.1 bar, frequency 120 Hz, temperature of 37 °C, and voltage of 500 V. A magnetic stirrer (IKA, Staufen, Germany) was placed in front of the encapsulator for the constant stirring of the gelling solution. The obtained beads were left in the gelling solution for 20 min after formation, rinsed with distilled water and filtered through Whatman No. 40 filter paper (Whatman International Ltd., Kent, UK), after which they were frozen at −80 °C for 1 h. The beads were freeze-dried in a laboratory freeze-dryer (Christ, Osterode am Harz, Germany) with isothermal plate temperatures of 20 °C for 24 h under high vacuum (13–55 Pa), vacuumed sealed using a FoodSaver^®^ vacuum sealer (Sunbeam Products, Inc., Boca Raton, FL, USA), and stored at −18 °C in nitrogen gas atmosphere until further analysis. The process was carried out in duplicate.

### 2.6. Encapsulation Efficiency (EE)

For the determination of EE, the beads were dissolved in a 5% sodium citrate solution at a ratio of 1:100 after 4 h on a magnetic stirrer (IKA, Staufen, Germany) at room temperature. The TPC of the dissolved beads was determined according to the procedure described in Section 2.4 and expressed as mg GAE L^−1^ of the dissolved beads solution. The EE percentage was calculated using the following equation:

EE % = (TPC_B_/TPC_0_) × 100 (1)

where TPC_B_ is the TPC in the sodium citrate dissolved beads solution, and TPC_0_ is the TPC in the initial bay leaf extract (theoretical load) calculated through a mass balance method. The beads were dissolved and analyzed in a duplicate (*n* = 4).

### 2.7. Antioxidant Activity Assays

The antioxidant activity of the beads dissolved in 5% sodium citrate as described in Section 2.6 was analyzed via DPPH radical scavenging assay and Ferric Reducing Antioxidant Power (FRAP) assay as previously described [14]. For DPPH, 0.75 mL of extract was mixed with 1.5 mL of DPPH methanol solution (0.2 mM) and incubated for 20 min in the dark at 23 °C. 2.25 mL methanol was used as blank. For FRAP, 80 µL of extract, 240 µL of distilled water, and 2080 µL of FRAP regent (acetate buffer (pH 3.6):TPTZ (10 mM in 40 mM HCl):FeCl_3_ (20 mM in distilled water) in a ratio of 10:1:1) were mixed, vortexed and incubated for 5 min at 37 °C. The absorbances were read on a VWR UV-1600PC Spectrophotometer (VWR, Wayne, PA, USA) at 517 nm and 593 nm for DPPH and FRAP, respectively. The standard curves were produced using Trolox. All measurements were performed in duplicate, and the results were expressed as mean value ± standard deviation of µmol Trolox-equivalent (TE) g^−1^ beads.

### 2.8. UPLC-MS^2^

The polyphenolic content of the extracts and dissolved beads was determined using an Agilent 1290 RRLC UPLC-MS^2^ system coupled with 6430 Triple Quadrupole LC/MS mass spectrometer (Agilent, Santa Clara, CA, USA). The ionization with ESI source was performed in +/− ionization mode with nitrogen as a desolvation and collision gas. A 100 × 2.1 mm Zorbax Eclipse Plus C18 (Agilent, Santa Clara, CA, USA) with 1.8 µm particle size was used for separations at 35 °C with the injection volume 2.5 µL. Flow rate was set at 11 L h^−1^, nebulizer pressure, drying gas temperature, and capillary voltage at 40 psi, 300 °C, and 4000/−3500 V, respectively. The limits of detection and quantification as well as solvent composition and gradient were previously described [16]. The data analysis and instrument control were carried out using MassHunter Workstation (ver. B.04.01) software (Agilent, Santa Clara, CA, USA). Identification and quantitative determination of individual polyphenols were described in detail in our previous research [17]. The polyphenols concentrations were expressed as mg L^−1^ of the extract or solution (mean value ± standard deviation). The analyses were carried out in duplicate.

### 2.9. Release Kinetics of Polyphenols

For the determination of BLP’s release kinetics in water, 300 mg of the freeze-dried alginate beads were suspended in 10 mL of distilled water and continuously agitated at 100 rpm on a shaker (IKA, Staufen, Germany) at room temperature. Every 10 min, an aliquot was taken from the supernatant and replaced by the same volume of distilled water. The TPC of the aliquots was determined as described in Section 2.4, and the results were expressed as mg GAE g^−1^ beads. The experiments were performed in a duplicate. 

The experimental data from the release kinetics study were fitted to a Korsmeyers–Peppas model using Microsoft Office Excel ver. 2019 according to the equation:

(2) $$f_{t}=\frac{M_{t}}{M_{\infty }}=K\times t^{n}$$

where *M_t_* and $M_{\infty }$ stand for the content of released polyphenols at time *t* and infinity, respectively. The $M_{\infty }$ can be considered the content of polyphenols in the initial beads. *K* represents the release velocity coefficient, while *n* is the release exponent indicating the release mechanism, including Fickian diffusion (*n* < 0.43), non-Fickian (anomalous) transport (0.43 < *n* < 0.85), and the super case II transport mechanism (*n* > 0.85) [18]. 

### 2.10. Bioaccessibility of Polyphenols

The bioaccessibility of the encapsulated BLP was examined according to a recently described three-step in vitro model [19], with modifications. Briefly, 200 mg of freeze-dried beads (750 μL of bay leaf extract) was placed in 50 mL reaction tubes and mixed with 800 μL of 0.1 M HCl pepsin solution (40 mg mL^−1^) and 10 mL of 0.9% sodium chloride solution. The pH was adjusted to 2 with 0.1 M HCl in required volume if necessary. For the gastric phase of digestion, the samples were shaken at 100 rpm in a water bath for 1 h at 37 °C. The reaction was stopped by placing the reaction tubes on ice for 5 min, and 1 mL of 0.9% NaCl and 1 mL of 0.5 M NaHCO_3_ were added into Pur-A-Lyzer 6–8 kDa dialysis membranes (Sigma-Aldrich, Steinheim, Germany), which were then placed in the reaction tubes, and the incubation was continued for 45 min to simulate the transition from stomach to the small intestine. Afterwards, the pH was adjusted to 6.5 by adding 1 M NaHCO_3_ in the required volume, and reaction was continued after adding 2.5 mL of pancreatin–bile salts solution (2 mg mL^−1^/12 mg mL^−1^). The samples were returned to the water bath at 37 °C at 100 rpm for 2 h to simulate the intestinal phase, after which they were put on ice to stop the reaction. An aliquot of 2 mL was taken from each phase and filtered through 0.45 μm syringe filters into glass vials for the UPLC-MS^2^ analysis of the phenolic content. The samples were stored at −18 °C in in nitrogen gas atmosphere. The process was carried out in duplicate. 

### 2.11. Statistical Analysis

Statistical analysis was performed in the Statistica ver. 12.0 (Statsoft Inc., Tulsa, OK, USA) software. For the determination of optimal encapsulation conditions, TPC of the beads, DPPH, and FRAP values were the variables dependent on the alginate percentage and type of gelling solution, whose influence was evaluated through a full factorial design comprising 36 trials. Shapiro–Wilk and Levene’s tests were applied to analyze the normality of the data sets and the homoscedasticity of the data sets’ variance. One-way and multifactorial analysis of variance (ANOVA) and Tukey’s HSD post hoc multiple comparison test were applied to normally distributed data, while the data which were not normally distributed and/or homogenic were analyzed using nonparametric Kruskal–Wallis one-way ANOVA and multiple comparison of mean ranks. All of the tests were considered significant at *p* ≤ 0.05. 

## 3. Results and Discussion

### 3.1. Influence of the Encapsulation Parameters on the Phenolic Content and Antioxidant Activity of BLP

This study examined the influence of alginate percentage and type of gelling solution on the phenolic content of the encapsulated bay leaf extracts as well as antioxidant activity determined by DPPH and FRAP according to the full factorial design shown in Table 1. In order to exclude the influence of alginate and gelling solution on the results of spectrophotometric analysis, blank beads were produced and analyzed. Phenolic compounds or antioxidant activity were not detected in the blank beads.

As shown in Table 1, the EE was in the range of 44.56–98.30%, while the antioxidant activity determined by DPPH and FRAP ranged from 10.63–20.18 μmol TE g^−1^ and 7.14–19.18 μmol TE g^−1^ bead, respectively. These results show that different conditions significantly influence the examined parameters, showing the importance of optimization processes. The raw data were statistically analyzed, and the results are shown in Table 2.

As can be observed in Table 2, the percentage of alginate had no statistically significant influence on any of the dependent variables indicating that 1% is enough to achieve efficient entrapment of BLP. The gelling solution significantly influenced (*p* < 0.01) all the dependent variables. Generally, higher EE and antioxidant activity were achieved when the gelling solutions containing chitosan were applied. This can be explained by the improvement of alginate porous structure in the presence of other polysaccharides, such as chitosan, which enables higher EE, namely of lower molecular polyphenols [20]. In addition, the lower pH of the gelling solution containing chitosan might have influenced the interaction between the mannuronic and glucuronic acid (pK 3.38 and 3.65, respectively) in alginate with different groups of polyphenols, ensuring the chemical entrapment of polyphenols in the bead’s matrix since it was suggested that the protonated form of alginate shows greater ability to bind phenolic compounds than the deprotonated form [21]. The highest EE, as well as antioxidant activity determined using both FRAP and DPPH, were achieved when 1.5% CaCl_2_ with 0.5% chitosan was used as a gelling solution. Further increase in CaCl_2_ percentage resulted in lower values in the presence of chitosan, while there was no statistically significant influence of gelling solution when CaCl_2_ solutions without chitosan were used, indicating that 1.5% CaCl_2_ is adequate to form calcium alginate beads with maximum EE and antioxidant activity of BLP. The decrease in the values in the presence of chitosan might be due to the interaction of calcium ions from 3% and 5% CaCl_2_ solutions with the amino groups of chitosan molecule, leading to less amino groups available for the binding of polyphenols [22,23]. 

Based on the results of statistical analysis, 1% alginate and 1.5% CaCl_2_ + 0.5% *w*/*v* chitosan gelling solution (S4) were chosen as optimal for obtaining maximum EE and antioxidant activity of the encapsulated extracts. Under these conditions, the achieved EE was 92.76%, which is higher than the 73.76% achieved via encapsulation in nanoliposome [5] and in the range of 72.9–99.3% achieved through spray-drying [4], indicating that electrostatic extrusion is an efficient technique for the encapsulation of BLP. The optimal sample was further analyzed for individual phenolic compounds using UPLC-MS^2^ and compared to the sample obtained by using 1% alginate and 1.5% CaCl_2_ (S1) in order to observe the influence of chitosan presence and the difference in the pH of the gelling solution. The content of individual phenolic compounds was analyzed in the initial extract as well, and theoretical load was calculated for each compound through mass balance in order to estimate the EE for each of the detected compounds. The results are shown in Table 3. 

Flavonols were the most abundant group of polyphenols in the initial extract as well as in S1 and S4, where quercetin glycosides (mainly quercetin-3-glucoside) were the main representatives. Phenolic acids (dominantly caffeic acid) were also present in large portions, while flavan-3-ols, proanthocyanidins, and flavones were present in significantly lower quantities. Differences were observed in the TPC determined by spectrophotometric analysis and the results of UPLC-MS^2^ that can be explained by the presence of other compounds such as organic acids, sugars, and chlorophyll present in the bay leaf extract, which interact with the Folin–Ciocalteu reagent, leading to seemingly higher values of TPC [24,25]. The results of UPLC-MS^2^ confirmed the results of spectrophotometric analysis, which showed that gelling solution containing chitosan results in a higher EE of total polyphenols. However, differences were observed in the EE of individual polyphenols and polyphenolic groups. While the EE for phenolic acids was not affected by the gelling solution, higher percentages of flavones and flavonols were encapsulated with the use of gelling solution containing chitosan (S4). On the other hand, higher percentages of flavan-3-ols and proanthocyanidins were encapsulated in absence of chitosan (S1). These differences imply that the entrapment of polyphenols may be affected by the specific structures of individual compounds and by their various moieties (i.e., carboxylic and hydroxylic groups) and molecular masses, resulting in different binding affinities for the alginate and chitosan functional groups. In addition, some polyphenols could interact with the uronate and glucosamine residues of alginate chains, explaining the selective bonding of certain polyphenols [8].

### 3.2. Release Kinetics of BLP

**Figure 1:**
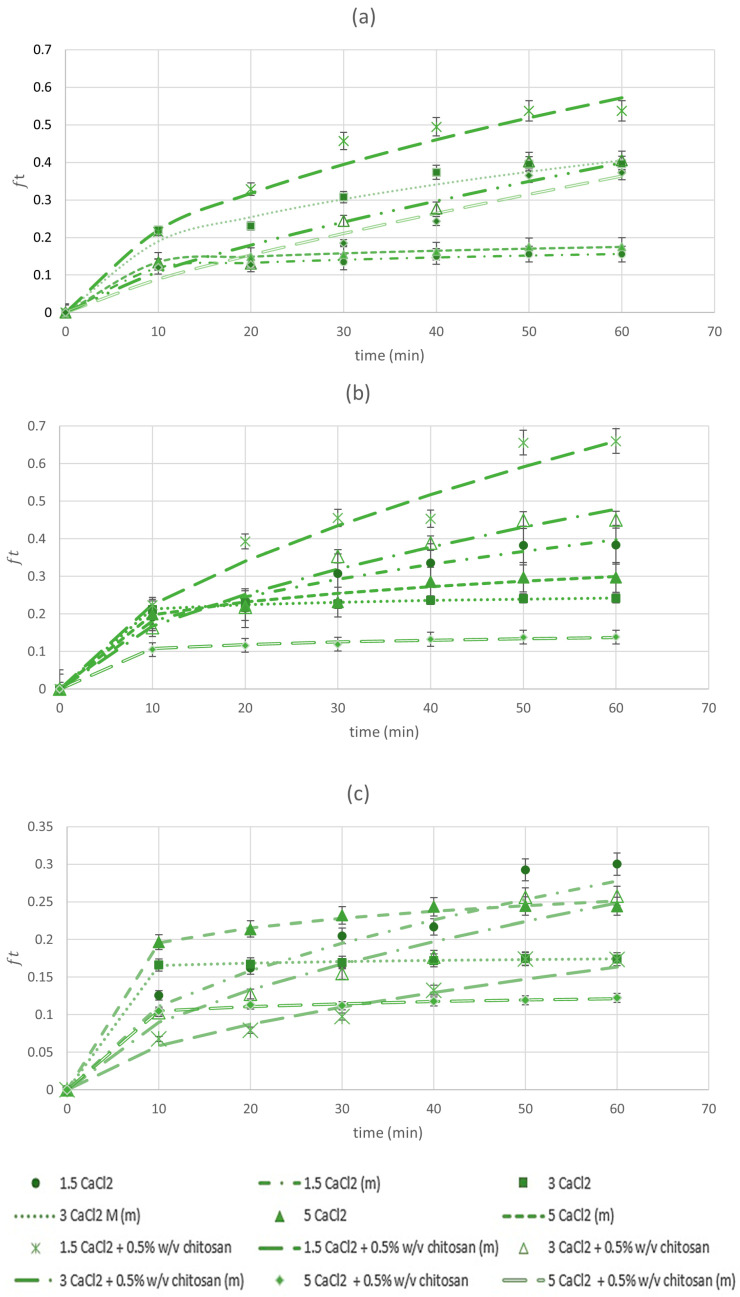
Plots of fraction of bay leaf polyphenols released in water versus time and modelling for the correlation of kinetic parameters: (**a**) 1.0% alginate; (**b**) 1.5% alginate, (**c**) 2% alginate. Data are expressed as mean ± standard deviation. Symbols: experimental data; lines: modelling results. (m) = model.

The release profile of BLP from the beads produced using 1% alginate (Figure 1a), 1.5% alginate (Figure 1b), and 2% alginate (Figure 1c) and all combinations of gelling solutions were investigated after placing the beads in water and measuring the TPC of the surrounding medium in selected intervals during 60 min. The obtained data on TPC were fitted according to the Korsmeyer–Peppas model equation (Equation (2)). For all data sets, the correlation coefficient R^2^ was higher than 0.95 indicating a good correlation between the model and experimental data. Release velocity coefficient K and release exponent *n* were determined from the data fitting (Table 4).

Over 60 min, the highest level of release of polyphenols from 1% and 1.5% alginate beads was obtained using 1.5% CaCl_2_ + 0.5% chitosan, and the lowest in the sample was obtained using 1.5% CaCl_2_ and 5% CaCl_2_ + 0.5% chitosan, respectively, as gelling solutions. The latter gelling solution resulted in the lowest release level in 2% alginate beads as well, while the highest level in the case was obtained by 1.5% CaCl_2_. These results indicate that chitosan combined with the highest percentage of CaCl_2_ results in the lowest release level of BLP at alginate percentage 1.5% or higher, most likely due to enhanced alginate–chitosan complexation and alginate cross-linkage, which resulted in the increase in the beads’ mechanical strength and consequently lower water penetration [26]. Even though the level of release was higher in some beads obtained by gelling solutions with chitosan, the values of release velocity coefficient *K* showed that these beads generally released BLP at a lower rate than those without chitosan, meaning that they reached equilibrium later than samples obtained without chitosan in gelling solution. This is consistent with previous data on slower release of polyphenols in water from alginate beads reinforced with chitosan [8,9]. 

The values of release exponent *n* were in the range of 0.07–0.79, indicating that different mechanisms were involved in the release of BLP depending on the conditions under which the beads were produced. The release mechanism of the most beads obtained using gelling solutions without chitosan was consistent with Fickian diffusion (*n* < 0.43), while the release of polyphenols from most beads obtained using gelling solutions with chitosan was controlled by multiple mechanisms (0.43 < *n* < 0.85), including diffusion, swelling, and erosion of the polymeric matrix [18]. According to the kinetic parameters *K* and *n*, the beads which showed the lowest levels of release (1.5% or 2% alginate with 5% CaCl_2_ + 0.5% chitosan) also had relatively low release rates and followed Fickian diffusion, which indicates that beads obtained at these parameters behave in the most predictable manner, which makes them the most suitable for application in the food industry.

### 3.3. Bioaccessibility of BLP

**Figure 2:**
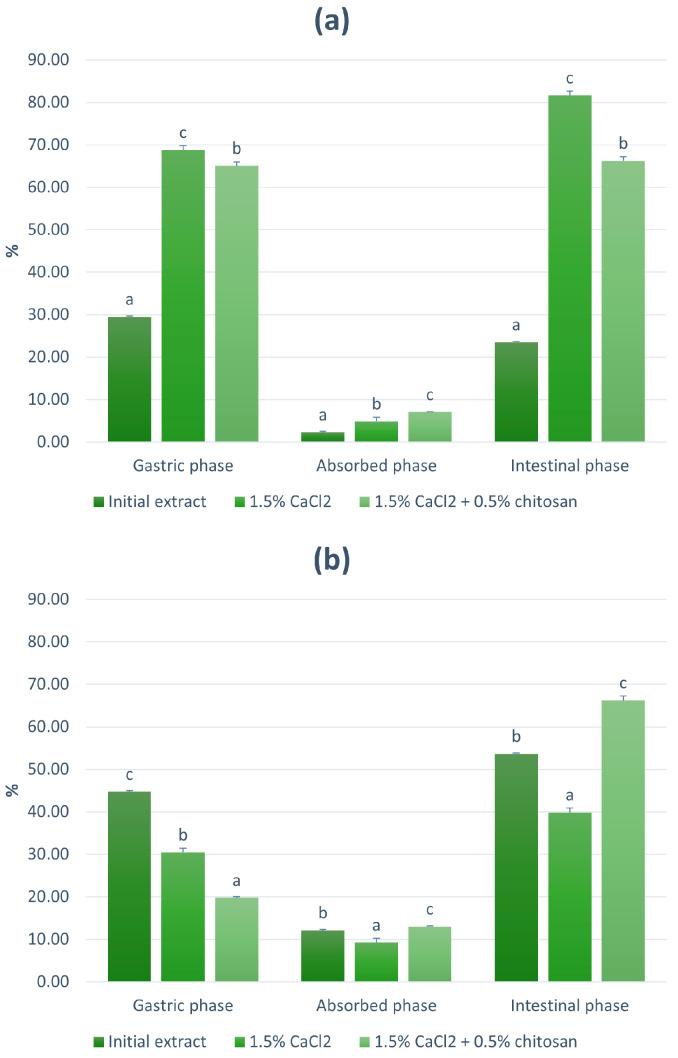
Bioaccessibility profile of (**a**) quercetin-3-glucoside and (**b**) caffeic acid from the initial extract and encapsulated extracts using 1% alginate. Values with different letters are statistically different at *p* ≤ 0.05.

The bioavailability of polyphenols depends on their release during different stages of digestion (bioaccessibility), which is highly influenced by the food matrix or—in case of microencapsulation—the applied carrier [27]. In order to observe the influence of encapsulation on the bioaccessibility of BLP, the most abundant compounds (quercetin-3-glucoside and caffeic acid) from the initial extract, S1 (1% alginate + 1.5% CaCl_2_), and S4 (1% alginate + 1.5%CaCl_2_ + 0.5% chitosan) were analyzed using UPLC-MS^2^ and monitored through three stages of in vitro digestion. As expected, encapsulation generally increased the bioaccessibility of both quercetin-3-glucoside (Figure 2a) and caffeic acid (Figure 2b). During the gastric phase of digestion, the bioaccessible percentage of quercetin-3-glucoside was twice as high in S1 and S4 as in the initial extract, showing that encapsulation protected this compound from the hydrolysis in the acidic environment, which occurs in different ratios depending on the food matrix [28]. On the other hand, caffeic acid had the highest bioaccessible percentage from the initial extract, possibly due to its relative stability in the acidic gastric environment [29]. The release of caffeic acid from the polymeric matrices was lower than in the intestinal phase, in which the structural change of alginate and chitosan caused by the change in pH from acidic to neutral possibly resulted in higher release of both caffeic acid and quercetin-3-glucoside [30,31]. Encapsulation using the gelling solution containing chitosan allowed the highest absorption of both compounds, which mirrors the findings in vivo, where—in addition to the sustained stability of polyphenols [10]—chitosan can also enhance the absorption due to its mucoadhesive properties, which allow longer presence in the small intestine and consequently higher absorption [32]. The absorbed percentage of caffeic acid (13.01%) was higher than that of quercetin-3-glucoside (7.07%), which is in agreement with the literature data, which reported the absorption of quercetin and its glycosides to be lower than 10% [33], while the absorption of caffeic acid can reach the percentage of 19.1% [34]. 

These results provide an insight into the bioccessibility of the main BLP, which can be useful in formulating food products with the encapsulated bay leaf extract. Further research of the colon phase of digestion and the interaction with the gut microbiota, which plays a major role in the polyphenols’ metabolism [27], as well as following the fate of metabolites in plasma would give further insight into the bioavailability of BLP and their fate and biological activity in the human body.

## 4. Conclusions

This study demonstrated for the first time that electrostatic extrusion in an alginate-based system can be considered as an efficient technique for the encapsulation of BLP. The encapsulation efficiency, antioxidant activity, and release kinetics largely depended on the applied encapsulation parameters. The combination of 1% alginate and 1.5% CaCl_2_ with 0.5% chitosan as a gelling solution resulted in the highest encapsulation efficiency and antioxidant activity in vitro. The use of 1.5% or 2% alginate with 5% CaCl_2_+ 0.5% chitosan as a gelling solution resulted in the most controlled release of BLP. Generally, encapsulation increased the bioaccessibility of BLP and the presence of chitosan in the gelling solution showed potential for higher absorption of the main BLP representatives, quercetin-3-glucoside, and caffeic acid. These results indicate that the combination of calcium alginate with chitosan generally results in more desirable properties of the obtained beads, showing the highest potential for use in functional food products. In order to achieve maximum usage of the potential of BLP, further research should be focused on varying the alginate–chitosan ratio along with other encapsulation conditions, taking into account the characteristics and proposed application purpose of the encapsulated BLP.

## Figures and Tables

**Table 1:** Total phenolic content, encapsulation efficiency, and antioxidant activity of bay leaf polyphenols encapsulated under different conditions.

Sample	% Alginate	Gelling Solution	Total Phenols mg GAE/g Bead	Encapsulation Efficiency %	DPPH μmol TE/g Bead	FRAP μmol TE/g Bead
1	1	1.5% CaCl_2_	10.94 ± 0.28	52.44 ± 1.36	10.63 ± 0.94	9.62 ± 0.11
2	1	3% CaCl_2_	12.31 ± 0.46	59.35 ± 2.23	11.88 ± 0.72	11.12 ± 0.59
3	1	5% CaCl_2_	10.46 ± 0.16	50.25 ± 0.77	11.66 ± 0.71	8.33 ± 0.38
4	1	1.5% CaCl_2_ + 0.5% chitosan	19.22 ± 0.78	92.76 ± 3.78	19.47 ± 0.23	16.96 ± 1.3
5	1	3% CaCl_2_ + 0.5% chitosan	12.68 ± 0.64	60.92 ± 3.1	16.01 ± 0.16	12.12 ± 0.43
6	1	5% CaCl_2_ + 0.5% chitosan	10.68 ± 0.40	51.2 ± 1.94	14.18 ± 0.21	9.95 ± 0.27
7	1.5	1.5% CaCl_2_	12.11 ± 1.03	58.12 ± 4.94	11.78 ± 0.37	10.68 ± 0.11
8	1.5	3% CaCl_2_	9.82 ± 0.04	47.37 ± 0.19	10.49 ± 0.77	7.14 ± 0.38
9	1.5	5% CaCl_2_	10.33 ± 0.58	50.04 ± 2.81	12.27 ± 0.05	8.15 ± 0.75
10	1.5	1.5% CaCl_2_ + 0.5% chitosan	19.85 ± 0.93	95.43 ± 4.45	20.18 ± 0.63	17.03 ± 1.19
11	1.5	3% CaCl_2_ + 0.5% chitosan	13.82 ± 0.69	66.26 ± 3.29	17.42 ± 1.11	14.03 ± 0.92
12	1.5	5% CaCl_2_ + 0.5% chitosan	12.33 ± 0.40	59.14 ± 1.94	16.01 ± 0.58	10.14 ± 0.33
13	2	1.5% CaCl_2_	10.22 ± 0.83	49.08 ± 3.97	11.74 ± 0.85	11.59 ± 0.43
14	2	3% CaCl_2_	9.21 ± 0.54	44.56 ± 2.61	11.76 ± 0.44	11.27 ± 0.65
15	2	5% CaCl_2_	10.33 ± 0.60	49.97 ± 2.9	12.12 ± 0.35	12.19 ± 0.97
16	2	1.5% CaCl_2_ + 0.5% chitosan	20.38 ± 0.68	98.3 ± 3.29	19.14 ± 0.32	19.18 ± 1.08
17	2	3% CaCl_2_ + 0.5% chitosan	15.15 ± 1.11	72.49 ± 5.32	16.87 ± 0.04	16.29 ± 0.38
18	2	5% CaCl_2_ + 0.5% chitosan	9.22 ± 0.36	44.77 ± 1.74	14.4 ± 0.38	11.46 ± 0.11

Results are expressed as mean ± standard deviation.

**Table 2:** Influence of encapsulation parameters on total phenolic content, encapsulation efficiency, and antioxidant activity of bay leaf polyphenols.

	N	Total Phenols	EE (%)	DPPH	FRAP
		(mg GAE g^−1^ Beads)		(μmol TE g^−1^ Beads)	(μmol TE g^−1^ Beads)
% alginate		*p* = 0.41 ‡	*p* = 0.39 ‡	*p* = 0.87 ‡	*p* = 0.06 ‡
1	12	12.71 ± 0.92 ^a^	61.15 ± 4.46 ^a^	13.97 ± 0.92 ^a^	11.35 ± 0.85 ^a^
1.5	12	13.04 ± 1.01 ^a^	62.73 ± 4.84 ^a^	14.69 ± 1.05 ^a^	11.19 ± 1.03 ^a^
2	12	12.42 ± 1.24 ^a^	59.86 ± 5.97 ^a^	14.34 ± 0.86 ^a^	13.66 ± 0.92 ^a^
Gelling solution		*p* ≤ 0.01 †	*p* ≤ 0.01 †	*p* ≤ 0.01 †	*p* ≤ 0.01 †
1.5% CaCl_2_	6	11.09 ± 0.42 ^a^	53.21 ± 2.04 ^a^	11.38 ± 0.34 ^a^	10.63 ± 0.37 ^a^
3% CaCl_2_	6	10.44 ± 0.61 ^a^	50.43 ± 2.94 ^a^	11.38 ± 0.35 ^a^	9.84 ± 0.87 ^a^
5% CaCl_2_	6	10.37 ± 0.16 ^a^	50.09 ± 0.75 ^a^	12.02 ± 0.18 ^a^	9.55 ± 0.87 ^a^
1.5% CaCl_2_ + 0.5% *w*/*v* chitosan	6	19.82 ± 0.33 ^c^	95.49 ± 1.59 ^c^	19.60 ± 0.24 ^c^	17.72 ± 0.59 ^c^
3% CaCl_2_ + 0.5% *w*/*v* chitosan	6	13.89 ± 0.52 ^b^	66.56 ± 2.47 ^b^	16.77 ± 0.33 ^b^	14.15 ± 0.79 ^b^
5% CaCl_2_ + 0.5% *w*/*v* chitosan	6	10.74 ± 0.58 ^a^	51.71 ± 2.70 ^a^	14.86 ± 0.39 ^b^	10.52 ± 0.31 ^a^
Average	36	12.72 ± 0.60	61.25 ± 2.88	14.33 ± 0.53	12.07 ± 0.56

Results are expressed as mean ± SE. † Statistically significant at *p* ≤ 0.05. ‡ Statistically insignificant at *p* > 0.05. Values with different letters within the same column are statistically different at *p* ≤ 0.05. EE = encapsulation efficiency.

**Table 3:** Identification and encapsulation efficiency of individual BLP as determined by UPLC-MS^2^.

Compound Number	Retention Time	Tentative Identification	Concentration (mg L^−1^)					
			Extract	Bead Extract Theoretical	Bead Extract Experimental		EE (%)	
					S1	S4	S1	S4
Phenolic acids								
2	3.745	3,4-dihidrobenzoic acid hexoside	0.19 ± 0.01	0.02 ± 0.00	0.00 ± 0.00	0.00 ± 0.00	23.49 ± 1.33 ^b^	5.82 ± 0.33 ^a^
3	4.55	Protocatehuic acid	5.41 ± 0.15	0.54 ± 0.02	0.25 ± 0.01	0.29± 0.01	45.69 ± 2.59 ^a^	53.75 ± 3.04 ^b^
4	4.79	Syringic Acid	8.62 ± 0.24	0.86 ± 0.02	0.64 ± 0.02	0.66 ± 0.02	74.11 ± 4.19 ^a^	76.97 ± 4.36 ^a^
5	4.913	Chlorogenic acid	0.97 ± 0.03	0.10 ± 0.00	0.06 ± 0.00	0.07 ± 0.00	60.61 ± 3.43 ^a^	72.20 ± 4.09 ^b^
6	5.43	Rosmarinic acid	1.65 ± 0.05	0.17 ± 0.00	0.16 ± 0.00	0.10 ± 0.00	98.58 ± 5.58 ^b^	61.20 ± 3.46 ^a^
7	6.492	Caffeic acid	119.74 ± 3.39	11.97 ± 0.34	5.03 ± 0.14	3.87 ± 0.11	42.02 ± 2.38 ^b^	32.30 ± 1.83 ^a^
12	7.931	*p*-coumaric acid	4.13 ± 0.12	0.41 ± 0.01	0.25 ± 0.01	0.36 ± 0.01	59.85 ± 3.39 ^a^	87.32 ± 4.94 ^b^
17	8.568	Ferulic acid	2.76 ± 0.08	0.28 ± 0.01	0.24 ± 0.01	0.27 ± 0.01	85.21 ± 4.82 ^a^	98.03 ± 5.55 ^b^
24	9.76	*p*-hydroxybenzoic acid	4.10 ± 0.12	0.41 ± 0.01	0.34 ± 0.00	0.41 ± 0.01	83.03 ± 3.48 ^a^	99.19 ± 4.15 ^b^
28	11.443	Gallic acid	5.73 ± 0.16	0.57 ± 0.02	0.55 ± 0.02	0.26 ± 0.01	95.65 ± 5.41 ^b^	46.15 ± 2.61 ^a^
-	-	∑Phenolic acids	153.30 ± 0.43	15.33 ± 0.04	7.51 ± 0.02	6.30 ± 0.02	49.02 ± 3.66 ^a^	41.07 ± 3.44 ^a^
Flavonols								
1	3.604	Kaempferol-3-*O*-rutinoside	24.44 ± 0.69	2.44 ± 0.07	2.36 ± 0.07	2.32± 0.07	96.57 ± 5.47 ^a^	94.86 ± 5.37 ^a^
15	8.343	Rutin	78.43 ± 2.22	7.84 ± 0.22	5.76 ± 0.16	4.44± 0.13	73.45 ± 4.16 ^b^	56.58 ± 3.20 ^a^
18	8.62	Quercetin-3-*O*-glucoside	108.39 ± 3.07	10.84 ± 0.31	7.56 ± 0.21	10.70± 0.30	69.78 ± 3.95 ^a^	98.74 ± 5.59 ^b^
19	9.161	Kaempferol-3-*O*-hexoside	27.27± 0.77	2.73 ± 0.08	1.43 ± 0.04	1.50 ± 0.04	52.44 ± 2.97 ^a^	54.84 ± 3.10 ^a^
20	9.171	Quercetin-3-*O*-pentoside	19.35 ± 0.55	1.93 ± 0.05	1.06 ± 0.03	1.77 ± 0.05	54.83 ± 3.10 ^a^	91.71 ± 5.19 ^b^
22	9.528	Isorhamnetin3-*O*-hexoside	40.85 ± 1.16	4.08 ± 0.12	2.86 ± 0.08	4.95 ± 0.07	69.97 ± 3.96 ^a^	121.21 ± 1.65 ^b^
23	9.548	Quercetin-3-*O*-rhamnoside	60.23± 1.70	6.02 ± 0.17	4.98 ± 0.14	6.00 ± 0.17	82.73 ± 4.68 ^a^	99.64 ± 5.64 ^b^
25	9.829	Kaempferol-3-*O*-pentoside	6.02± 0.17	0.60 ±0.02	0.51 ± 0.01	0.58 ± 0.02	84.64 ± 4.79 ^a^	96.98 ± 5.49 ^b^
27	10.346	Kaempferol-3-*O*-deoxyhexoside	0.14± 0.00	0.01 ± 0.00	0.01 ± 0.00	0.01 ± 0.00	40.02 ± 2.26 ^a^	63.15 ± 3.57 ^b^
29	12.176	Myricetin	0.85 ± 0.02	0.09 ± 0.00	0.08 ± 0.00	0.08 ± 0.00	98.18 ± 5.56 ^a^	99.69 ± 5.64 ^a^
-	-	∑Flavonols	365.96 ± 1.04	36.60 ± 0.1	26.61 ± 0.08	32.36 ± 0.08	72.73 ± 4.09 ^a^	88.42 ± 4.44 ^b^
Flavones								
11	7.589	Luteolin-6-*C*-glucoside	0.31± 0.01	0.03 ± 0.00	0.01 ± 0.00	0.03 ± 0.00	46.97 ± 2.66 ^a^	86.95 ± 4.92 ^b^
14	8.223	Apigenin-6-*C*-(*O*-deoxyhexosyl)-hexoside	0.14 ± 0.00	0.01 ± 0.00	0.00 ± 0.00	0.00 ± 0.00	15.28 ± 0.86 ^a^	30.78 ± 0.42 ^b^
21	9.261	Luteolin	8.62 ± 0.24	0.86 ± 0.02	0.54 ± 0.02	0.86 ± 0.02	62.80 ± 3.55 ^a^	99.61 ± 5.64 ^b^
26	10.24	Apigenin	1.07 ± 0.03	0.11 ± 0.00	0.02 ± 0.00	0.03 ± 0.00	21.02 ± 1.19 ^a^	28.98 ± 1.64 ^b^
		∑Flavones	10.14 ± 0.07	1.04 ± 0.00	0.58 ± 0.02	0.92 ± 0.03	57.25 ± 2.07 ^a^	90.82 ± 3.15 ^b^
Flavan-3-ols								
8	6.581	Catechin	14.50 ± 0.41	1.45 ± 0.04	0.34 ± 0.01	0.14 ± 0.00	23.14 ± 1.31 ^b^	9.73 ± 0.55 ^a^
9	6.588	Epicatechin	14.90 ± 0.42	1.49 ± 0.04	0.33 ± 0.01	0.16 ± 0.00	22.27 ± 1.26 ^b^	10.77 ± 0.61 ^a^
13	7.993	Epicatechin gallate	1.01 ± 0.03	0.10 ± 0.00	0.03 ± 0.00	0.04 ± 0.00	31.14 ± 1.76 ^a^	36.19 ± 2.05 ^b^
16	8.363	Epigallocatechin gallate	2.24 ± 0.06	0.22 ± 0.01	0.03 ± 0.00	0.07 ± 0.00	12.11 ± 0.69 ^a^	31.26 ± 1.77 ^b^
		∑Flavan-3-ols	32.65 ± 0.23	3.27 ± 0.02	0.73 ± 0.02	0.41 ± 0.02	22.24 ± 1.25 ^b^	12.50 ± 1.24 ^a^
Proanthocyanidins								
10	6.9	Procyanidin trimer	24.22 ± 0.68	2.42 ± 0.07	0.31 ± 0.01	0.15 ± 0.00	12.65 ± 0.72 ^b^	6.38 ± 0.36 ^a^
-	-	Total phenols	586.26 ± 2.46	58.63 ± 0.25	35.74 ± 0.31	40.41 ±0.45	60.97 ± 1.15 ^a^	68.46 ± 1.37 ^b^

Results are expressed as mean ± SD. Values with different letters in the same row are statistically different at *p* ≤ 0.05. S1 = 1% alginate and 1.5% CaCl_2_ gelling solution; S4 = 1% alginate and 1.5% CaCl_2_ + 0.5% *w*/*v* chitosan gelling solution. EE = encapsulation efficiency.

**Table 4:** The kinetic parameters for the release kinetics of encapsulated bay leaf polyphenols correlated using the Korsmeyers–Peppas model.

Sample	Alginate %	Gelling Solution	K	*n*	R^2^
1	1% alginate	1.5% CaCl_2_	0.0846	0.1499	0.9604
2		3% CaCl_2_	0.0714	0.4242	0.9606
3		5% CaCl_2_	0.0962	0.1469	0.9831
4		1.5% CaCl_2_ + 0.5% chitosan	0.0638	0.5357	0.9680
5		3% CaCl_2_ + 0.5% chitosan	0.0204	0.7260	0.9605
6		5% CaCl_2_ + 0.5% chitosan	0.0146	0.7851	0.9582
7	1.5% alginate	1.5% CaCl_2_	0.0657	0.4396	0.9752
8		3% CaCl_2_	0.1811	0.0717	0.9578
9		5% CaCl_2_	0.1156	0.2328	0.9530
10		1.5% CaCl_2_ + 0.5% chitosan	0.0562	0.6016	0.9611
11		3% CaCl_2_ + 0.5% chitosan	0.0434	0.5866	0.9747
12		5% CaCl_2_ + 0.5% chitosan	0.0787	0.1367	0.9768
13	2% alginate	1.5% CaCl_2_	0.0345	0.5091	0.9747
14		3% CaCl_2_	0.1545	0.0296	0.9531
15		5% CaCl_2_	0.1426	0.1382	0.9746
16		1.5% CaCl_2_ + 0.5% chitosan	0.0158	0.5704	0.9618
17		3% CaCl_2_ + 0.5% chitosan	0.0246	0.5646	0.9564
18		5% CaCl_2_ + 0.5% chitosan	0.0871	0.0813	0.9674

K = release velocity coefficient; *n* = release exponent; R^2^ = correlation coefficient.

## Data Availability

The data used to support the findings of this study can be made available by the corresponding author upon request.
